# Genome-wide investigation and expression analysis of sweet cherry *PavNAC* gene family under different abiotic stress

**DOI:** 10.3389/fpls.2025.1712680

**Published:** 2025-11-24

**Authors:** Xiaomeng Shi, Xianyang Ai, Changping Tian, Aidi Zhang, Jianzhao Li, Shuyang Sun

**Affiliations:** 1 School of Horticulture, Ludong University, Yantai, China; 2 Yantai Academy of Agricultural Sciences, Yantai, China; 3 School of Food Engineering, Ludong University, Yantai, China; 4 Yantai Technology Center of Characteristic Plant Gene Editing and Germplasm Innovation, Ludong University, Yantai, China

**Keywords:** sweet cherry, NAC transcription factors, expression analysis, abiotic stress, genome wide analysis

## Abstract

The *NAC* gene family is an important transcription factor unique to plants, widely involved in plant growth, development, and response to different stress. The physicochemical properties, phylogenetics, chromosome localization, gene structure and motif, collinearity, and gene expression under stress of the *PavNAC* transcription factor family in sweet cherry were analyzed. The results showed that the whole genome sequence of sweet cherry contains 132 *PavNAC* transcription factor genes, which are unevenly distributed on 8 chromosomes and divided into 10 subfamilies. The protein length of *PavNAC* ranged from 152 to 755 amino acids, and the CDS length ranged from 459 to 2268. The gene structure showed that most *PavNAC* members contain 3 exons and 10 motifs. Collinearity analysis result showed that 54 *PavNAC* genes in sweet cherry have homologous genes in *Arabidopsis*. Transcriptome data of different development stages of ‘Hong Deng’ and ‘Black Pearl’ showed that *PavNAC42* and *PavNAC101* were specifically expressed in both varieties. Finally, expression of 15 *PavNAC* genes under different abiotic stress were analyzed by real-time PCR. The results showed that *PavNAC70*, *PavNAC9*, and *PavNAC10* were induced by low temperature. *PavNAC70* and *PavNAC81* were both induced by salt and PEG stresses. The study laid the foundation for subsequent molecular breeding and further exploration of the function of *PavNAC* genes in sweet cherry.

## Introduction

Transcription factors are proteins that control the transcription rate of genetic information from DNA to messenger RNA (Latchman, 1997). They play a role by combining with cis acting promoter elements, and participate in the response of plants to abiotic stress such as drought, high salt, and low temperature (Liu et al., 2014; Zhang et al., 2016). According to its DNA domain, transcription factors in plants can be divided into several gene families, such as WRKY, bZIP, MYB, DREB, AP2/ERF, C2H2, NAC, etc (Nogueira et al., 2005; Singh et al., 2021). These genes typically function collectively as members of large families, and individual members within a family can participate in different stress responses.

The NAC transcription factor gene family is one of the largest transcription factor families unique to plants (Gao et al., 2020; Lin et al., 2023). The NAC protein structure consists of a highly conserved DNA binding domain located at the N-terminus and a highly variable transcriptional regulatory region located at the C-terminus (Nie et al., 2020). Among them, the N-terminal is further divided into five sub domains: A, B, C, D, and E, which contain nearly 160 amino acid residues (Zhang et al., 2016). Among them, the sub domains A, C, and D are highly conserved, while the B and E domains vary among different plants (Ooka et al., 2003; Olsen et al., 2005). At the same time, the C-terminal exhibits high diversity in amino acid composition and function. With the continuous progress and improvement of bioinformatics technology, more and more plant NAC families have been identified and studied. For example, researchers have found 117 NAC proteins in Arabidopsis, 151 NAC proteins in rice (Nuruzzaman et al., 2010), 102 NAC proteins in cocoa beans (Shen et al., 2020), and 104 NAC proteins in medium grain tomato (Su et al., 2014).

The NAC gene plays multiple functions in plants (Puranik et al., 2012), including regulating the development of flowers and leaves (Vroemen et al., 2003; Aida et al., 1997), thickening of secondary cell walls (Zhong et al., 2006), protein and lipid metabolism pathways (Chi et al., 2017), leaf senescence and fruit development (Ma et al., 2018), lateral root formation (Xie et al., 2000), seed germination (Kim et al., 2008), and playing a role in plant senescence (Balazadeh et al., 2011). Researches have also found that NAC transcription factors play an important role in the response of plants to different stress. The NAC transcription factor region is rich in stress response elements, such as low temperature response elements, water deficiency response elements, damage response elements (Nakashima et al., 2012). The *OsNAC2* gene in rice can interact with ABA, enhancing its ability to resist drought and high salt stress (Jiang et al., 2019). *PtrNAC72* in goji berries can respond to drought stress by regulating proline (Wu et al., 2016). *StNAC053* in potatoes can regulate related genes to increase the activity of superoxide dismutase (SOD), catalase (CAT), and peroxidase (POD), thereby enhancing their drought tolerance (Wan F. X. et al., 2021). Under low temperature stress, banana *MaNAC1* can interact with ICE-CBF, thereby improving the cold resistance (Shan et al., 2014). The *SsNAC23* gene in sugarcane can also participate in response to low temperature stress (Nogueira et al., 2005). Under high salt stress, *TsNAC1* in salt mustard binds to a proton transporter protein to regulate plant salt tolerance (Liu et al., 2017).

Sweet cherry (*Prunus avium* L.) is a deciduous tree of the *Rosaceae* family, native to Europe and western Asia. It is widely cultivated in the mountainous areas of eastern China and also in temperate regions around the world. Sweet cherry is also one of the famous cultivated cherry varieties, with large fruit shape, beautiful flavor, rich nutrition, and containing various nutrients and bioactive components, such as glucose, vitamin C, and anthocyanins (Gao and Mazza, 1995). In agricultural production, the normal development of sweet cherry is a key factor in enhancing yield and influencing cultivation expansion. However, sweet cherry is highly vulnerable to low-temperature frost damage during the flowering and early fruit stages, leading to reduced fruit yield and diminished quality. With a relatively shallow root system, sweet cherry is also susceptible to significant fruit drop when water supply is insufficient during development, such as under drought conditions, resulting in economic losses. Additionally, sweet cherry is extremely sensitive to salt stress, and soil salinization further restricts its cultivation. Therefore, studying their growth and development mechanisms is particularly important.

However, little is known about the NAC gene family in sweet cherry, particularly its roles in stress resistance. In order to better understand the molecular regulation mechanism of sweet cherry during development, it is necessary to study the NAC gene family in sweet cherry. The NAC family members were identified within the entire genome of sweet cherry, and their physicochemical properties, phylogenetic relationships, gene structure, conserved motifs, and chromosomal localization were analyzed. Furthermore, we analyzed the expression levels of selected *PavNAC* genes at different developmental stages in two sweet cherry cultivars, ‘Black Pearl’ and ‘Hong Deng’, using transcriptome data, as well as the expression of *PavNAC* genes under abiotic stress conditions, including low temperature, drought, and high salt. Finally, expression of 15 *PavNAC* genes under different abiotic stress were analyzed by q-PCR.

## Materials and methods

### Plant materials

The experimental materials used in this experiment were 3-month-old sweet cherry tissue cultured seedlings from the sweet cherry germplasm resource bank of Yantai academy of agricultural sciences. Transcriptome analysis was performed on two sweet cherry cultivars, ‘Hong Deng’ and ‘Black Pearl’. 200 mM NaCl was added to the MS medium for NaCl treatment. 20% PEG6000 (w/v) was added to MS medium for PEG treatment. MS medium was used as the control group. Sweet cherry seedlings were transplanted and propagated *in vitro* and cultured under normal conditions for 1, 2, 3, 4, and 5 days. Select 15 healthy seedlings with normal growth status and place them into a low-temperature (LT, 4°C) incubator for 1, 2, 3, 4, and 5 days as low-temperature treatment. The control group seedlings were grown in a room temperature (RT, 24 ± 1°C) growth chamber. The experiment was performed with three independent biological replicates for each of the five treatment groups. Finally, these seedlings were frozen in liquid nitrogen and stored at - 80°C for RNA extraction and gene expression analysis in subsequent experiments.

### Identification of PavNAC genes family

The protein, CDS and genome sequence of sweet cherry were obtained from NCBI database (http://www.ncbi.nlm.nih.gov). The PF02365 of the NAC domain was download from Pfam database (http://pfam.xfam.org). Then, HMMER was used to find the possible NAC transcription factor family from sweet cherry genome. In order to verify whether the existing family members are accurate, the online software SMART tool (http://smart.embl-heidelberg.de) was used to confirm the specific number of NAC gene family members in sweet cherry by analyzing its domain.

### Phylogenetic analysis of the PavNAC genes

In order to further study and analyze the evolutionary relationship between NAC families. The “One Step Build a ML Tree” function in TBtools software was used to build an evolutionary tree containing 132 PavNAC and 105 Arabidopsis NAC full-length protein sequences. According to the known classification information of Arabidopsis NAC gene family and phylogenetic tree analysis, sweet cherry NAC family was classified into different subgroups.

### Chromosomal distribution of PavNAC genes

The starting and ending positions of candidate genes are obtained through the general feature format (GFF) annotation file extracted from the sweet cherry genome, and they are renamed according to their positions on the chromosome. The “Gene Location from GTF/GFF” function in TBtools software was used to make the location map of genes on the chromosome, where Chr width ratio was set to 0.03, and the color and font were modified and adjusted automatically. The settings of other default parameters remain unchanged.

### Gene structures and motif analyses of PavNAC genes

In order to further understand the gene structure of *PavNAC*s, the intron and exon composition of all gene sequences were analyzed by comparing with genome sequences. The “Visualize Gene Structure” function in TBtools software was used to analyze gene structure, drag the sweet cherry genome annotation file (GFF) and the gene ID of the PavNAC family to draw a domain analysis diagram of the PavNAC gene family. The online tool MEME (http://meme-suite.org/tools/meme) was used to find the conserved motif. The motif discovery function is used to predict and analyze the conserved motifs of 132 NACs. The number of software parameter motifs is set to 10, and default values are used for other parameters. In addition, TBtools visualization can also be used to combine evolutionary tree files with gene structure data to form composite graphs.

### Collinearity analysis of PavNAC genes

To further explore the common origin of the genome, we used TBtools software to analyze the linear relationship between sweet cherry and Arabidopsis species. The “one-step MCScanX” function of TBtools was used, the genome sequence files and gene structure annotation files of sweet cherry and Arabidopsis were dragged to the corresponding boxes to start, and the process was completed when TBtools displayed “completion”. Then the “DuaI Systeny Plot” function to pull the Ctl, GFF, and collinearity files obtained in the previous step into the corresponding boxes based on the file type, and enter the gene ID of the PavNAC gene family to start the plot.

### RNA extraction and quantitative real-time PCR

RNAprep pure plant plus kit (Tiangen, Beijing, China) was used to isolate total RNA for qPCR analysis. The integrity of RNA was detected by agarose gel electrophoresis, and the concentration of RNA was detected by spectrophotometer. Hiscript III RT supermax (Vazyme, Nanjing, China) was used to eliminate genomic DNA contamination and synthesize the first strand cDNA. RT-qPCR primers were designed using Primer3.0 software, with *PavActin* used as the reference gene, and their specificity was assessed using the NCBI BLAST program (**Supplementary Table S1**). ChamQ Universal SYBR qPCR Master Mix Kit (Vazyme, Nanjing, China) and bio-rad CFX connected real-time system were used to complete Q-PCR.

### Statistical analysis

In statistical analysis, LSD analysis after one-way ANOVA was used. The graphics were generated with GraphPad Prism 6 (GraphPad Software, San Diego, CA, USA).

## Results

### Identification of PavNAC genes family

BLAST alignment was performed on the identified sweet cherry NAC genes, then duplicate sequences were removed to ultimately identify 132 *PavNAC* genes, which were named *PavNAC001-PavNAC132* based on their positions on chromosomes (**Supplementary Table S2**). According to the analysis of various physicochemical properties of the PavNAC gene family, the number of amino acids in the PavNAC family members ranges from 152 (PavNAC122) to 755 (PavNAC86), with an average CDS length of around 1107 bp. The longest CDS length is 2268 bp, and the shortest is only 459 bp.

### Phylogenetic analysis of the PavNAC genes

**Figure 1:**
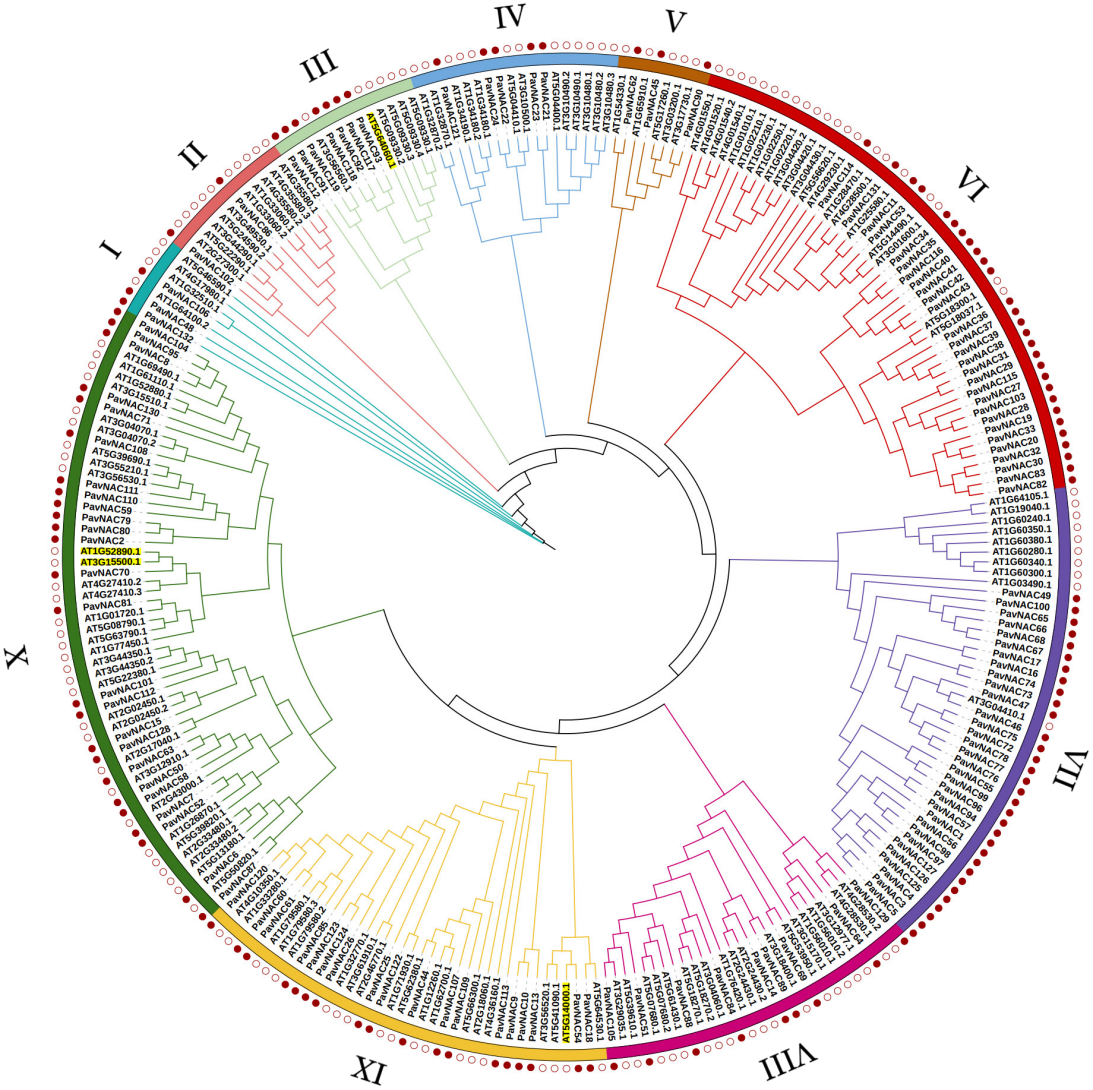
Phylogenetic analysis of NAC proteins in sweet cherry and Arabidopsis. 132 PavNAC transcription factor proteins from sweet cherry and 105 AtNAC proteins from Arabidopsis were selected to construct a rooted phylogenetic tree. This NAC evolutionary tree is divided into 10 different subgroups.

To further investigate the evolutionary relationship of PavNAC, 132 sweet cherry PavNAC transcription factor protein sequences and 105 AtNAC transcription factor protein sequences from Arabidopsis were used to construct a phylogenetic tree. Based on the known family member classification information in Arabidopsis NAC transcription factors, the sweet cherry NAC gene family was classified into ten subgroups (**Figure 1**). The X subgroup had the most branches and members. The VII and VI subgroups contain the most members of the PavNAC gene family, with 32 and 28, respectively. The I, II, and V subgroups contain the fewest members of the PavNAC gene family, with only 2-3. All subgroups contain both sweet cherry and Arabidopsis NAC proteins, indicating a certain degree of evolutionary similarity between the NAC families of the two species. The difference in the number of branches and branching genes among different subgroups indicated that there is still a difference in each subfamily even when the homologous domain is the same. These differences may lay the structural foundation for the functional diversity of the PavNAC gene family.

### Chromosome distribution of PavNAC genes

**Figure 2:**
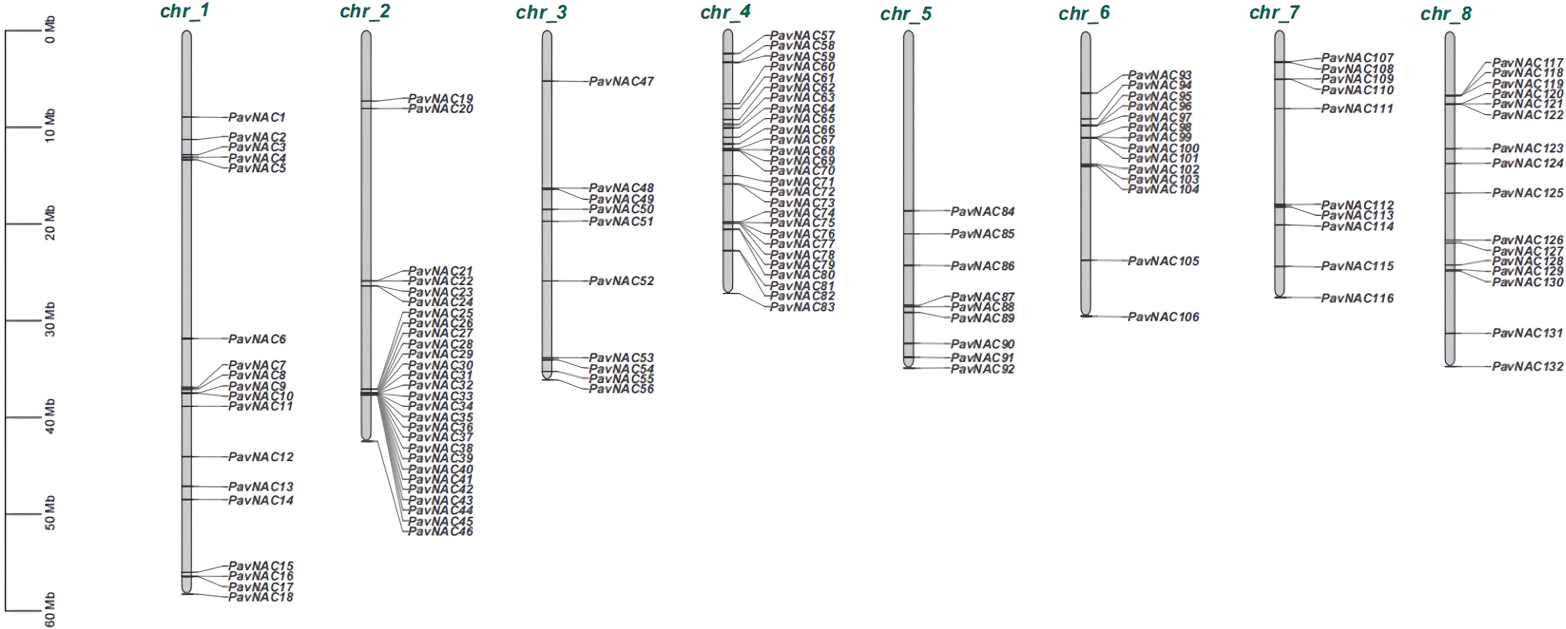
Localization of *PavNAC* genes on sweet cherry chromosomes. The left scale bar represents the length (Mb) of each chromosome. The green color represents 8 different chromosomes, and the black short line represents the distribution of the *PavNAC* genes on each chromosome.

132 PavNAC gene family members were distributed on all 8 sweet cherry chromosomes, but the number of distributions on each chromosome were different (**Figure 2**). Among them, the most members of the PavNAC gene family were distributed on chromosome 2, with a total of 28, while the least members of the PavNAC gene family were distributed on chromosome 5, with only 9. Eighteen members of the PavNAC gene family were distributed on chromosome 1. Twenty-seven members of the PavNAC gene family were distributed on chromosome 4, accounting for approximately 4.89% of the total genome. In addition, genes on the unified chromosome mostly belong to different subgroups in the evolutionary tree.

### Gene structures and motif analyses of PavNAC genes

**Figure 3:**
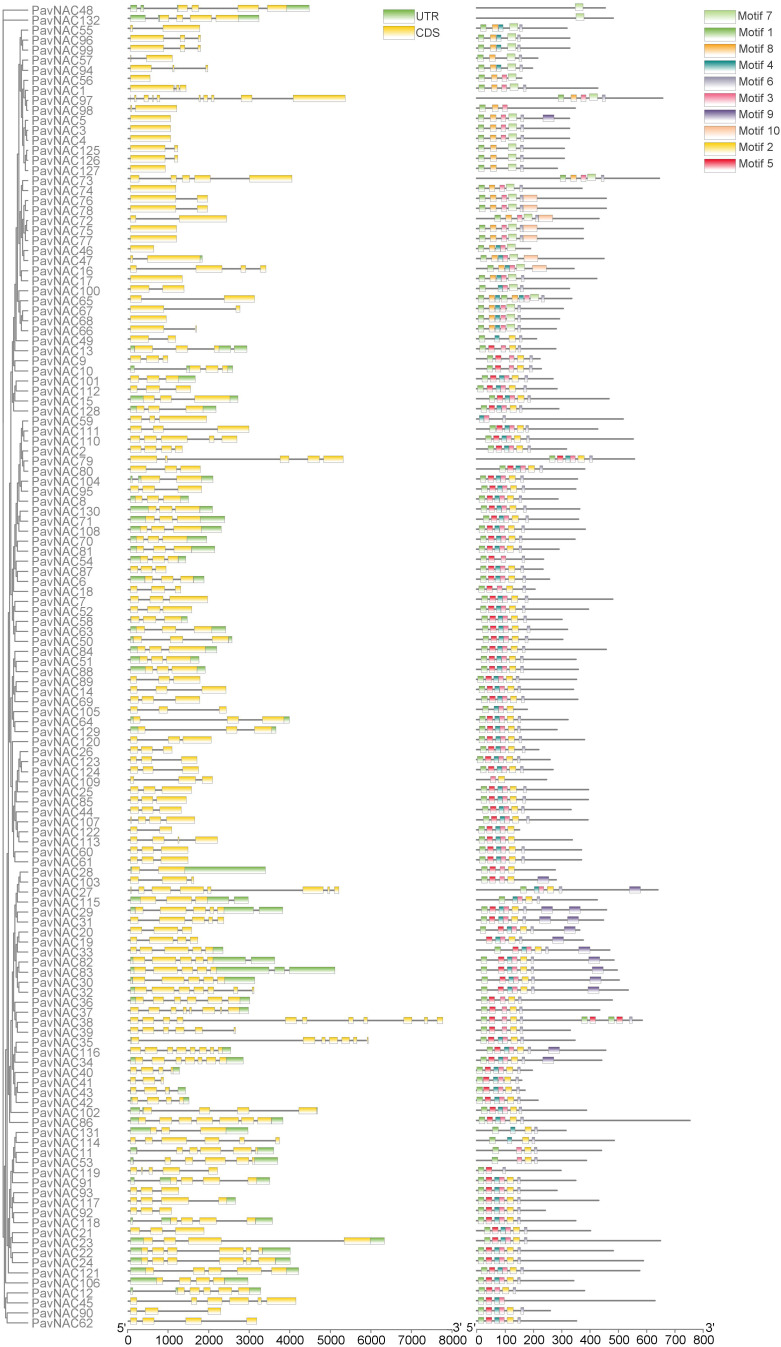
Phylogenetic relationship, motif analysis, and gene structure of *PavNAC* genes in sweet cherry. The left image is the phylogenetic tree of PavNAC proteins. The middle image represents the exon intron structure of the *PavNAC* genes. The black long line represents the length of the introns, and the yellow boxes represent the number of exons. The right image shows the conserved motifs in the PavNAC protein identified by MEME. The gray lines represent non conservative sequences, and the 10 motifs are represented by different colored boxes.

The introns and exons are crossed and embedded in a complete gene sequence, and each gene sequence contains an unequal number of introns and exons. Through the structural analysis of *PavNAC* family genes, it was found that the number of exons and introns in different subgroups were different, and the number in the same group was similar (**Figure 3**). According to the *PavNAC* structural analysis chart, the number of introns varies greatly, ranging from 0 to 10, and the number of exons ranges from 1 to 11. *PavNAC38* contains the most introns (10) and exons (11). Most PavNAC members contain three exons, which may be due to the relatively conservative domain of the NAC gene family (**Figure 3**). In order to further understand the structural characteristics of PavNAC gene family proteins, MEME software was used to analyze the conserved motifs of sweet cherry NAC proteins and obtained 10 motif information. It can be seen from the figure that motif 1, motif 2, motif 3, motif 4, motif 5 and motif 6 are present in more than 70% of sweet cherry PavNAC family members, and the family members containing motif 1, motif 3 and motif 6 are up to 90%. The motif sequence of most PavNAC members is: motif 1, motif 5, motif 4, motif 3, motif 2 and motif 6, indicating that PavNAC protein has similar motif composition. However, there are also a few PavNAC proteins with different motif composition.

### Collinearity analysis of PavNAC genes

**Figure 4:**
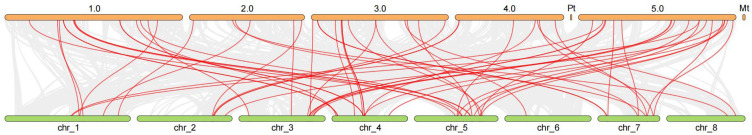
Homology analysis of NAC genes between sweet cherry and Arabidopsis. The red line represents the collinear relationship between sweet cherry and Arabidopsis. The orange bars at the top represent the five chromosomes of Arabidopsis, where Pt denotes the chloroplast genome and Mt denotes the mitochondrial genome of Arabidopsis. The green bars at the bottom represent the eight chromosomes of sweet cherry.

In order to further explore the evolution of PavNAC gene and the evolution relationship of NAC gene between different species, a collinearity analysis was carried out on Arabidopsis and sweet cherry (**Figure 4**). The result showed that there are 54 *PavNAC* genes with collinearity relationship between sweet cherry and Arabidopsis. In addition, the fifth chromosome in sweet cherry had the most homologous gene with Arabidopsis.

### HeatMap of PavNAC genes

**Figure 5:**
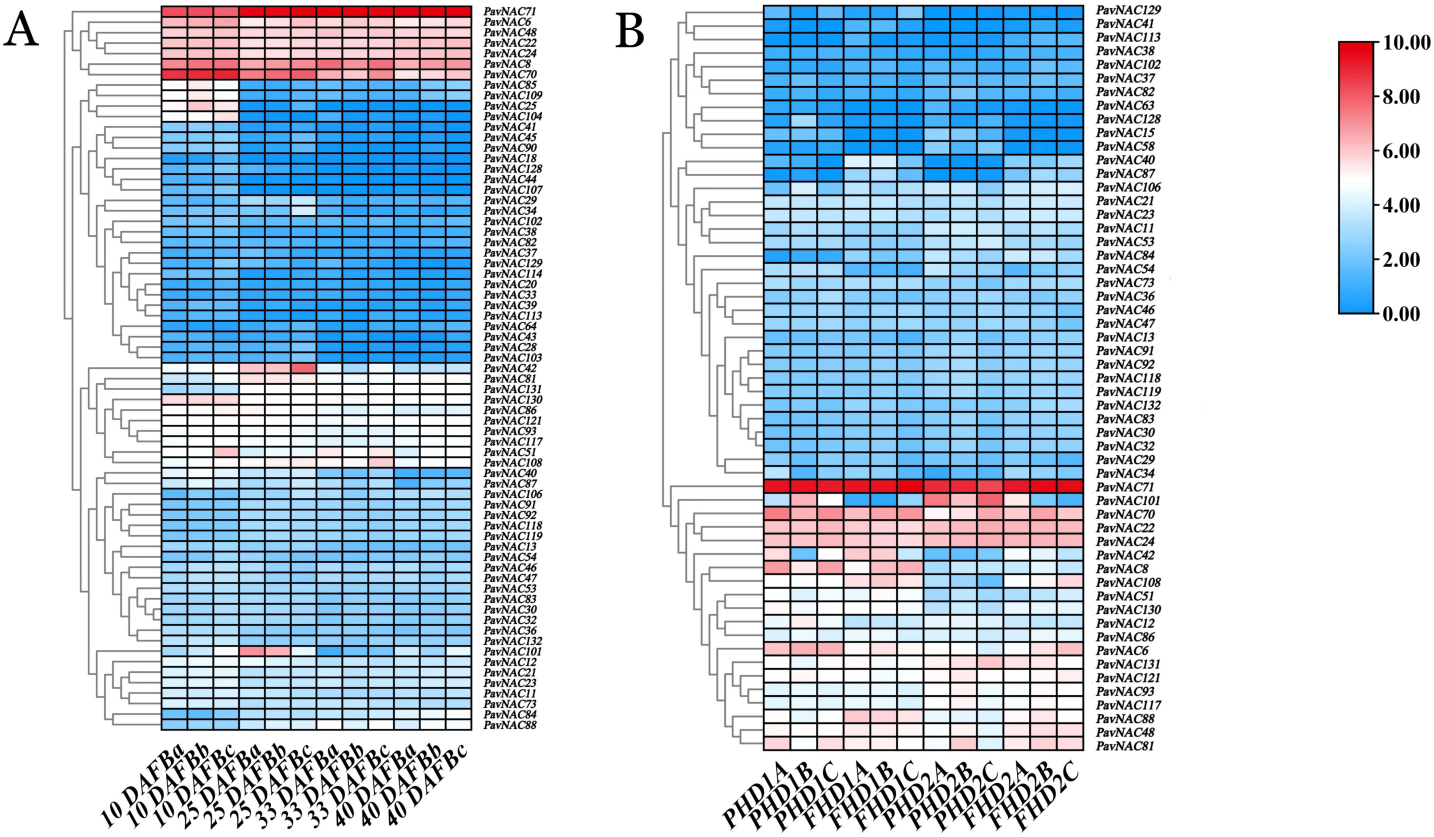
Heat map of *PavNAC* genes in different sweet cherry varieties. **(A)** The expression heat map of *PavNAC* genes in ‘Black Pearl’, from left to right, shows the different development stages at 10, 25, 33, and 40 DAFB. **(B)** Heat map of *PavNAC* genes in skin and flesh of ‘Hong Deng’. PHD1 stands for skin of 33 DAFB, FHD1 stands for flesh of 33 DAFB, PHD2 stands for skin of 48 DAFB, and FHD2 stands for flesh of 48 DAFB. The icon on the far right represents the relative expression value, with red indicating induction and blue indicating inhibition.

In order to study the expression characteristics of *PavNAC* genes during the growth and development of different sweet cherry cultivars, we conducted a study on the expression characteristics of 132 *PavNAC* gene family members in different tissues and organs by using the transcriptome data of ‘Black Pearl’ and ‘Hong Deng’ (**Figure 5**). During the fruit development of ‘Black Pearl’, the expression of *PavNAC71/6/48/22/24/8/70* showed a higher expression level than other detected genes (**Figure 5A**). The expression of *PavNAC71/22/24/70* showed a higher expression level than other detected genes in peel and flesh of ‘Hong Deng’ (**Figure 5B**).

### Expression of PavNAC genes under different abiotic stresses

**Figure 6:**
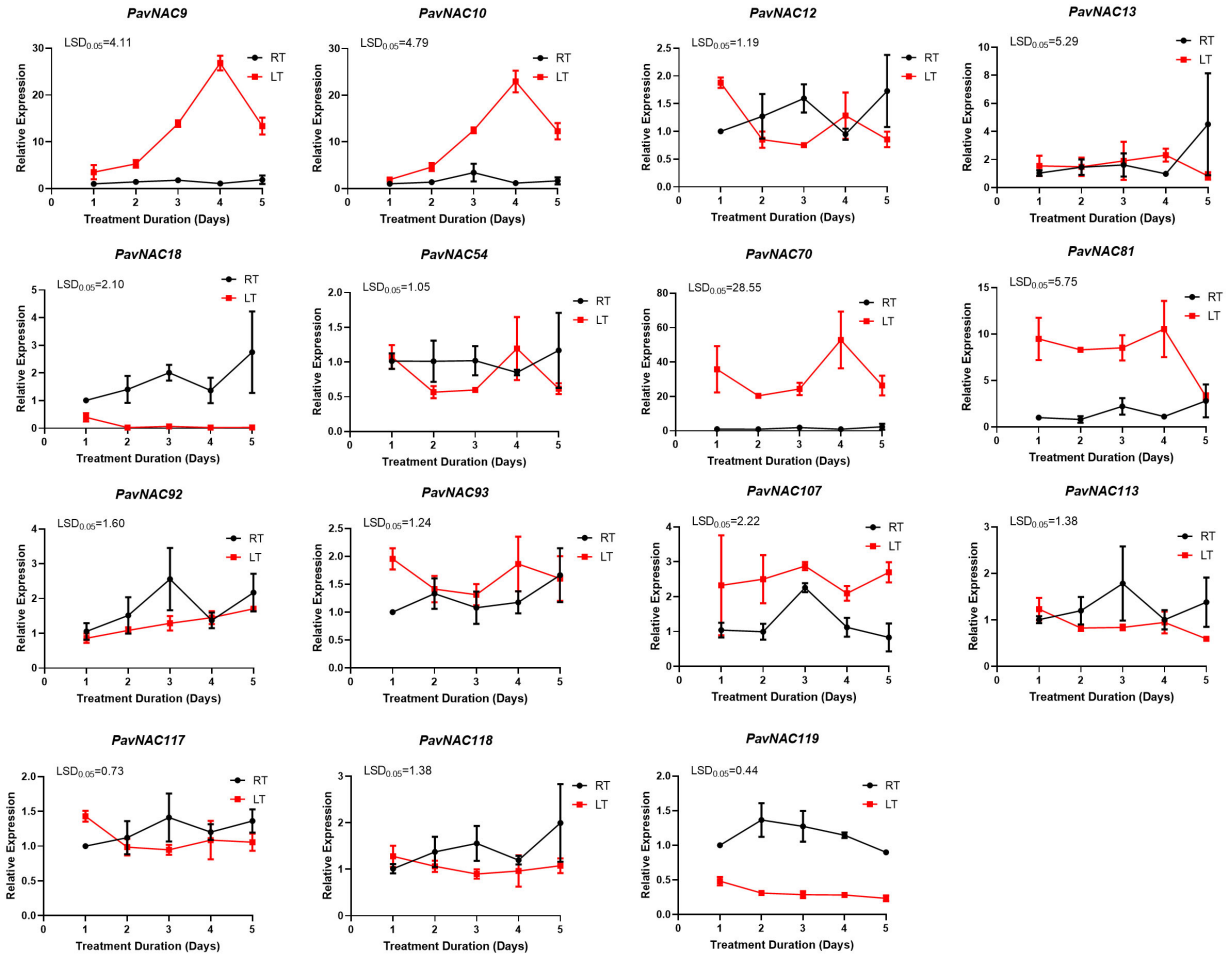
The relative expression level of *PavNAC* genes under low-temperature treatment. The black lines represent seedlings in the room temperature growth chamber as control. The red lines represent seedlings treated with low temperature. Data are presented as mean ± SD (n = 3). One-way ANOVA followed by Fisher’s LSD *post-hoc* test revealed statistically significant differences among the groups (*p* < 0.05).

**Figure 7:**
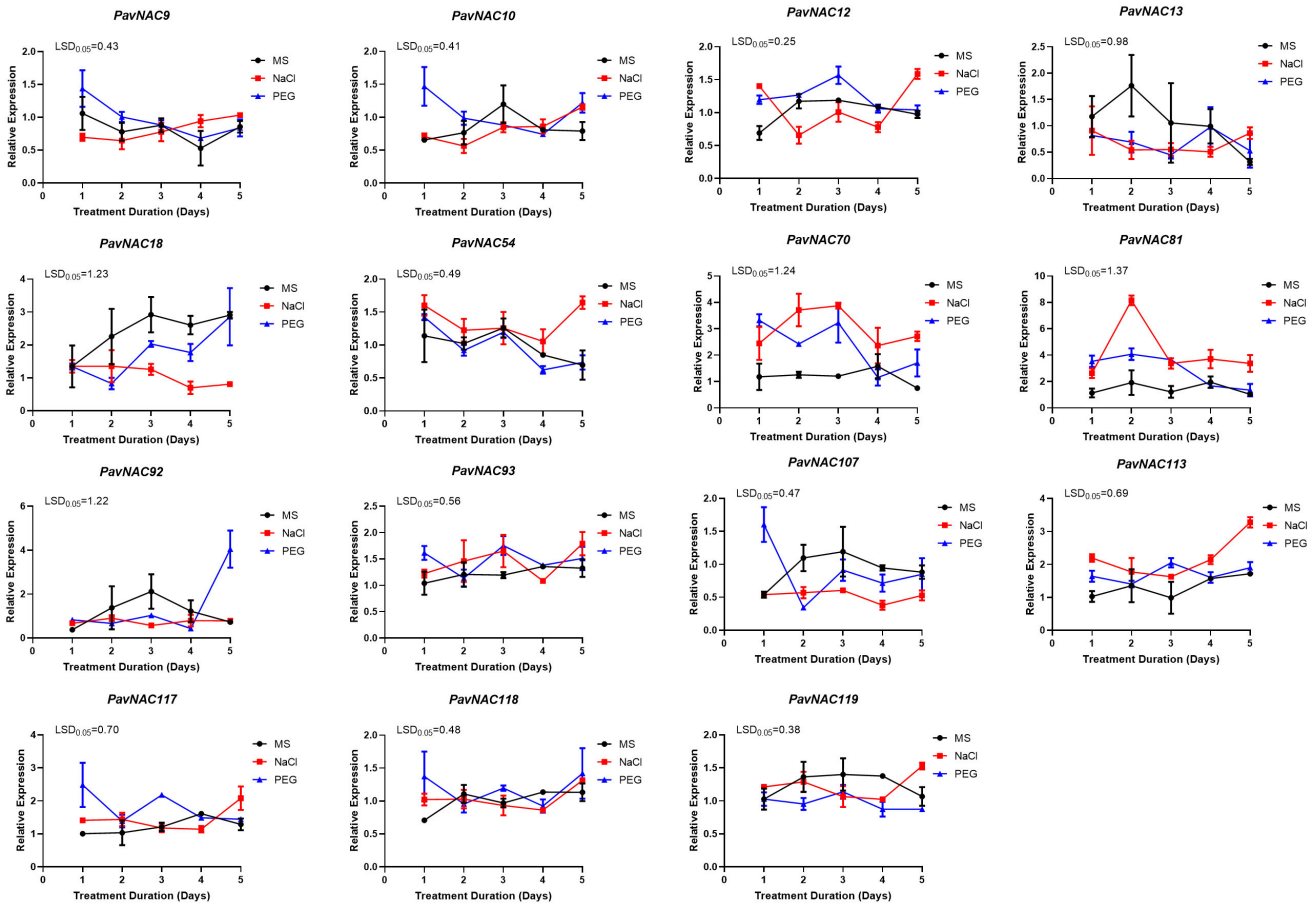
The relative expression levels of *PavNAC* genes under drought and salt stress. The black lines represent MS medium as the control group. The red and blue lines represent the culture medium treated with salt and PEG, respectively. Data are presented as mean ± SD (n = 3). One-way ANOVA followed by Fisher’s LSD *post-hoc* test revealed statistically significant differences among the groups (*p* < 0.05).

To investigate the regulatory roles of *NAC* family genes in the abiotic stress response of sweet cherry, we identified *PavNAC* genes that were either orthologs of known stress-responsive Arabidopsis *NAC* genes or clustered within the same phylogenetic clades. Based on this analysis, 15 *PavNAC* genes were selected for further study. Finally, 15 genes including *PavNAC9, PavNAC10,, PavNAC12, PavNAC13, PavNAC18, PavNAC54, PavNAC70, PavNAC81, PavNAC92, PavNAC93, PavNAC107, PavNAC113, PavNAC117, PavNAC118* and *PavNAC119* were quantitatively analyzed through qRT-PCR. The results showed that low temperature stress promoted the expression of most *PavNAC* genes (**Figure 6**). The expression levels of *PavNAC9*, *PavNAC10*, *PavNAC70* and *PavNAC81* under low temperature stress were higher than those under normal temperature, and they all showed a trend of first increasing and then decreasing, reaching the peak on the fourth day of stress. Among them, *PavNAC70* exhibited a markedly high expression level of 50, which corresponds to an approximately 50-fold upregulation relative to the control, under low-temperature stress at the fourth day. To further investigate the regulatory role of the *PavNAC* genes in response to high salt and PEG, quantitative analysis was conducted on these 15 genes. The results showed that most *PavNAC* genes were upregulated under high salt stress, with the peak expression of *PavNAC9*, *PavNAC13, PavNAC18, PavNAC70, PavNAC92, PavNAC93* and *PavNAC118* on the fifth day (**Figure 7**). *PavNAC93* showed the most significant expression response, with an expression level of up to 4000. High salt stress caused *PavNAC93* to show an expression value of approximately 4000, which is 4000-fold higher than the control. Under PEG treatment, most *PavNAC* genes showed a downward expression trend, with *PavNAC12, PavNAC13, PavNAC18, PavNAC54*, *PavNAC81, PavNAC107, PavNAC113* and *PavNAC119* being significantly expressed on the first day. Among these, *PavNAC18* and *PavNAC107* displayed the highest expression levels, reaching values of up to 100. It can be seen that different *PavNAC* genes in sweet cherry can exert physiological regulatory functions in different abiotic stresses.

## Discussion

There are various transcription factors in higher plants, many of which are related to their stress resistance (Gong et al., 2014). NAC transcription factors, as the largest class of plant specific transcription factors, can also enhance plant adaptability to stress (Li F. et al., 2019; Lin et al., 2023). NAC transcription factors are highly conserved at the N-terminus and highly differentiated at the C-terminus, which may enable the NAC gene family to control protein functions and affect plant growth and development (Olsen et al., 2005; Puranik et al., 2012). Although it has been identified in various plants such as Arabidopsis (Ooka et al., 2003), tomato (Su et al., 2014), celery (Duan et al., 2020), and rice (Meng et al., 2009), there is still a lack of research in sweet cherry.

This study conducted a genome-wide identification and analysis of the PavNAC transcription factor family in sweet cherry. The reliability of this research process primarily depended on high-quality genome assembly and precise genome annotation. As highlighted by Yang et al., the advent of high-quality long-read sequencing technologies and advanced assembly algorithms has propelled genome assembly into the telomere-to-telomere (T2T) era, establishing a robust foundation for the accurate identification of various gene family members (Yang et al., 2025). Building upon this resource, we successfully identified 132 *PavNAC* genes, effectively circumventing gene omission resulting from assembly gaps. Upon obtaining the complete gene set, precise gene annotation was crucial for ensuring the accuracy of all subsequent analyses. Lan et al. emphasized that annotation quality directly determines the accuracy of gene boundaries and intron-exon structures (Lan et al., 2025). In this study, both the phylogenetic analysis of *PavNAC* genes and the identification of conserved motifs relied on this accurate initial annotation. Furthermore, the selection of 15 key *PavNAC* candidate genes in sweet cherry for qRT-PCR validation also depended on the well-established genome annotations of *Arabidopsis thaliana*. In future research, integrating additional experimental evidence, such as long-read transcriptome data, will enable further refinement and expansion of the *PavNAC* gene models, thereby providing a solid foundation for investigating the regulatory functions of NAC transcription factors in abiotic stress resistance in sweet cherry.

Using the genome data of sweet cherry, this study identified 132 *PavNAC* genes, which were lower than that of rice (151 NAC genes) (Nuruzzaman et al., 2010), soybean (152 NAC genes) (Le et al., 2011) and tobacco (152 NAC genes) (Rushton et al., 2008), but higher than that of eggplant (49 NAC genes) (Wang et al., 2021) and Arabidopsis (117 NAC genes) (Nuruzzaman et al., 2010). The number of NAC gene family member in different species is different, which indicates that higher plants have experienced different types of gene replication in the process of evolution, and the number of similar gene families also indicates that they may have similar physiological functions. The NAC transcription factors of sweet cherry and *Arabidopsis* build a phylogenetic tree with 10 subgroups (**Figure 1**). The NAC transcription factors of Arabidopsis and sweet cherry are distributed in each subgroup, but there are differences in the number of NAC transcription factors of *Arabidopsis* and sweet cherry in each subgroup, indicating that the NAC family genes have the phenomenon of gene replication or loss in the long-term evolution process. Genes can be amplified in a variety of ways, such as genome replication, tandem replication, fragment replication and reverse locus replication (Wang et al., 2007). The results of chromosome mapping showed that PavNAC was widely distributed in sweet cherry genome, and there was a tandem repeat relationship, indicating that tandem repeat was an important amplification method of *PavNAC* gene in sweet cherry (**Figure 2**). In addition, the gene structure analysis showed that most PavNAC members contained two introns, and the conserved domains were mostly at the N-terminal, which was similar to the NAC gene structure in other plants (**Figure 3**). In general, the frequency of intron loss is higher than that of intron acquisition in the process of gene evolution. *PavNAC97* and *PavNAC38* contain more introns, and they may be the early members of *PavNAC*. PavNAC members belonging to the same subgroup have similar exon intron structures, which may be related to their similar functions. The results of domain analysis showed that in the same subgroup, PavNAC members contained the same number and order of motifs, and different subgroups had different motifs or specific motifs, which might play different roles. Motif 1, motif 3 and motif 6 were the most abundant among all PavNAC transcription factors. The results of collinearity analysis showed that the fifth chromosome was the most collinear chromosome with Arabidopsis thaliana, indicating that the *NAC* genes on this chromosome may retain orthologous relationships with those in Arabidopsis, making them strong candidates for functional studies based on known Arabidopsis gene functions (**Figure 4**).

The NAC gene family also plays an important role in promoting plant growth and development, such as leaf senescence, ovule development, seed coat formation, fruit ripening (Dong et al., 2019). The expression levels of the *PavNAC* gene family in two different varieties of sweet cherry at different flowering stages of ‘Black Pearl’ and in the skin and flesh of ‘Hong Deng’ (**Figure 5**). Heat map analysis shows that both *PavNAC42* and *PavNAC101* are highly expressed at 25 DAFB in ‘Black Pearl’. It is speculated that *PavNAC42* and *PavNAC110* are involved in the regulation of plant growth and development in the late flowering stage. PavNAC101 is significantly expressed in PHD compared to FHD in ‘Hong Deng’. The Arabidopsis NAC gene *AtNAC2*, which is homologous to *PavNAC101*, is involved in regulating the growth of thin-walled metastatic cell walls. Therefore, it is speculated that *PavNAC101* has a similar function (He et al., 2005; Nigarish et al., 2020).

In recent years, numerous transcription factor genes involved in plant responses to low temperature, drought, and high salinity have been investigated (Gong and Liu, 2013). To systematically examine the regulation of *PavNAC* gene expression under different abiotic stresses, this study also analyzed the effects of high salt, low temperature, and drought stress on the expression patterns of *PavNAC* genes. The control mechanisms of abiotic stress rely on the activation and regulation of a multitude of stress-related genes. Salt stress adversely affects plant growth and development by disrupting cellular osmotic balance and facilitating the accumulation of harmful substances (Zelm et al., 2020). Studies on the function of the NAC gene family in Arabidopsis have revealed that the *AtNAC019* protein can regulate the expression of cold stress-related genes by recognizing and binding to core DNA sequences (Jensen et al., 2010). In our research, we found that *PavNAC70*, which is homologous to *AtNAC019*, was highly expressed under low temperature stress, indicating that *PavNAC70* is also involved in the response to cold stress (**Figure 6**). Moreover, the sharp decline in the expression of most genes by the fourth day may suggest that low temperature leads to disruptions in physiological and metabolic activities. Transcriptional regulation constitutes a critical component of the plant response to low temperature stress (Li Y. Y. et al., 2019). In recent years, it has been progressively established that NAC transcription factors play significant roles in salt stress resistance. Research demonstrated that *ONAC022* functions as a stress-responsive NAC with transcriptional activator activity, playing a positive role in drought and salt stress tolerance by modulating ABA-mediated pathways (Hong et al., 2016). *AtNAC019, AtNAC055*, and *AtNAC072* are responsive to various abiotic stresses in Arabidopsis, including drought, low temperature, and high salinity (Tran et al., 2004). In our study, real-time PCR analysis revealed a particularly high expression level of *PavNAC93* in the high salt treatment group after four days (**Figure 7**). Furthermore, it was observed that different genes reached distinct peak expression levels at different time points, suggesting that the response of *PavNAC* genes to abiotic stress may also depend on the duration and intensity of the stress. The NAC transcription factor NTL4 (*AtNAC053*) promotes reactive oxygen species production during salt stress-induced leaf senescence in Arabidopsis, thereby mitigating the negative effects of high salinity. Given that *AtNAC053* is homologous to *PavNAC93* in the phylogenetic tree, it is hypothesized that *PavNAC93* may perform a similar function (Lee et al., 2012). The role of this gene in the stress response of sweet cherry warrants further investigation. Under drought stress, the expression of most genes exhibited a trend opposite to that under high salt stress, with high expression observed as early as the first day of PEG-simulated drought treatment. This indicates that the impact of high salt stress becomes more pronounced during later stages, underscoring the need for greater attention to the potential damage caused by salt stress in practical agricultural production. Three key *NAC* genes in Arabidopsis—*AtNAC019*, *AtNAC055*, and *AtNAC072*—have been confirmed to play important roles in drought stress response. *PavNAC81* in sweet cherry is homologous to these three Arabidopsis genes, suggesting that *PavNAC81* may perform a similar function in the drought stress response of sweet cherry. In addition to the 15 *PavNAC* genes investigated in this study, it is likely that numerous other genes are also involved in the stress response. Our findings further support the roles of *PavNAC* genes in responding to low temperature, drought, and high salinity stresses. Furthermore, by analyzing the phylogenetic relationship between sweet cherry *PavNAC* genes and *NAC* genes from other species, this study has laid the groundwork for further investigation into *NAC* gene functions.

## Conclusions

In this study, 132 *PavNAC* transcription factors in sweet cherry genome was identified. In addition, the expression patterns of *PavNAC* gene in tissues of different varieties were analyzed, and it was found that *PavNAC42* and *PavNAC101* were specifically expressed in ‘Black Pearl’ and ‘Hong Deng’ varieties, respectively. To verify the resistance of sweet cherry to abiotic stress, partial *PavNAC* genes were selected for qRT-PCR analysis. Research has found that *PavNAC70* is highly expressed under low temperature stress, while *PavNAC93* is highly expressed under high salt and PEG stress. These data will provide scientific basis for future research work, as well as excellent genetic resources for improving the stress resistance of sweet cherry through genetic engineering methods.

## Data Availability

The original contributions presented in the study are included in the article/**Supplementary Material**. Further inquiries can be directed to the corresponding authors.
