# Supplementary material for: Genome-wide investigation and expression analysis of sweet cherry PavNAC gene family under different abiotic stress
Source: Front Plant Sci. 2025 Nov 24;16:1712680. doi: 10.3389/fpls.2025.1712680 (PMC12682808; doi:10.3389/fpls.2025.1712680)
Supplement: Supplementary file 1 [file Table1.docx]

**Table S1** Primer sequences for RT-qPCR of *PavNAC* genes.

| Gene name | Forward primer (5' to 3') | Reverse primer(5' to 3') |  |  |
| --- | --- | --- | --- | --- |
| *PavNAC2* | TCCTCCCGCAAACTCTTCTT | GTCTTTGGCTGCGTCATTGA |  |  |
| *PavNAC9* | ACCTTCCCCACAATCGACTT | GGAAGTTCCCGGTCCAAAAT |  |  |
| *PavNAC10* | ACCTTCCCCACAATCGACTT | CCGGCCCAAAATCTTGTTGA |  |  |
| *PavNAC12* | TTGGTCATGTTTGCAGCCTC | GAGAAGTTGATGGGCCTCCT |  |  |
| *PavNAC13* | GAAAGGGAGGGATAGGGACG | GCACAGGACAAAAGAAGCCA |  |  |
| *PavNAC18* | TTAGGCGTTGAGGAACCAGT | CCTGCTGAGAGTGGAGAGAG |  |  |
| *PavNAC54* | ACTGGGTTGTGTATCGCGTA | TGGCATTGGAAGAGAGGTGA |  |  |
| *PavNAC59* | TCACTCCAGTCGAACGGAAA | GACGCCCAGCATAAAATCGT |  |  |
| *PavNAC70* | CAAGCTCGGGTTTCAGATGG | ATCAACCCGCTGAGTTCTGA |  |  |
| *PavNAC79* | AAATGGGGAAGGCATGGAGA | GGCACTTCATTCATGGGGAC |  |  |
| *PavNAC81* | CCGCCTTGCAAATGTCGATA | GGTGGTGGCATTTTGGTCAT |  |  |
| *PavNAC92* | GGTTTCGGTTCAAGCCAACT | TCAGATCCCCACTTTCCCAG |  |  |
| *PavNAC93* | CCCAACCGCTACTAATGCAC | CGACCTTCCGCCTCAATTTT |  |  |
| *PavNAC107* | AGGATGGGTTGTGTGTAGGG | GCTCTTGTTTGCACTGGGAA |  |  |
| *PavNAC110* | GCGGCTAGAAACAAGGATGG | TTATGGCCTCGGATAGCTGG |  |  |
| *PavNAC111* | TTGAGGCTTGCGTTCTATGC | GAGGAAGAAATCGGAGGGCT |  |  |
| *PavNAC113* | ACGGGTCGAGACAAATCAGT | GTAGCTTGAGTCCCACCCTT |  |  |
| *PavNAC117* | CCCAACCGCTACTAATGCAC | TTCTAAACGACCTTCCGCCT |  |  |
| *PavNAC118* | GGTTTCGGTTCAAGCCAACT | TCTCCACAATCCCAGCAAGA |  |  |
| *PavNAC119* | CCTGGTTTCCGGTTTTCTCC | CCACTTTGCCAGCCAGATTT |  |  |
| *PavActin* | CAACTATGTTCCCCGGTATTGC | CCCTTGGAAATCCACATCTGC |  |  |

**Table S2** Summary of the *PavNAC* gene family members information in sweet cherry.

| Number | Gene name | Gene ID | Chromosome | Start site | End site | CDS length | Protein length |
| --- | --- | --- | --- | --- | --- | --- | --- |
| 1 | *PavNAC1* | FUN_000668-T1 | chr_1 | 8937261 | 8938632 | 1290 | 429 |
| 2 | *PavNAC2* | FUN_000945-T1 | chr_1 | 11255803 | 11257082 | 957 | 318 |
| 3 | *PavNAC3* | FUN_001134-T1 | chr_1 | 12855667 | 12856656 | 990 | 329 |
| 4 | *PavNAC4* | FUN_001171-T1 | chr_1 | 13110489 | 13111478 | 990 | 329 |
| 5 | *PavNAC5* | FUN_001202-T1 | chr_1 | 13361623 | 13361623 | 990 | 329 |
| 6 | *PavNAC6* | FUN_003454-T1 | chr_1 | 31834203 | 31834203 | 777 | 258 |
| 7 | *PavNAC7* | FUN_004194-T1 | chr_1 | 36869292 | 36871195 | 1440 | 482 |
| 8 | *PavNAC8* | FUN_004229-T1 | chr_1 | 37070906 | 37072338 | 867 | 288 |
| 9 | *PavNAC9* | FUN_004309-T1 | chr_1 | 37072338 | 37072338 | 678 | 225 |
| 10 | *PavNAC10* | FUN_004310-T1 | chr_1 | 37472370 | 37474894 | 690 | 229 |
| 11 | *PavNAC11* | FUN_004522-T1 | chr_1 | 38831517 | 38835049 | 1329 | 442 |
| 12 | *PavNAC12* | FUN_005433-T1 | chr_1 | 44045420 | 44048631 | 1149 | 382 |
| 13 | *PavNAC13* | FUN_005957-T1 | chr_1 | 47107867 | 47110743 | 843 | 280 |
| 14 | *PavNAC14* | FUN_006180-T1 | chr_1 | 48470816 | 48473174 | 1032 | 343 |
| 15 | *PavNAC15* | FUN_007259-T1 | chr_1 | 55996756 | 55999408 | 1410 | 469 |
| 16 | *PavNAC16* | FUN_007313-T1 | chr_1 | 56403016 | 56406360 | 1038 | 345 |
| 17 | *PavNAC17* | FUN_007315-T1 | chr_1 | 56417509 | 56418786 | 1278 | 425 |
| 18 | *PavNAC18* | FUN_007582-T1 | chr_1 | 58253279 | 58254521 | 624 | 207 |
| 19 | *PavNAC19* | FUN_008120-T1 | chr_2 | 7275486 | 7277146 | 1137 | 378 |
| 20 | *PavNAC20* | FUN_008211-T1 | chr_2 | 8057111 | 8058620 | 1098 | 365 |
| 21 | *PavNAC21* | FUN_010208-T1 | chr_2 | 25853020 | 25854836 | 1212 | 403 |
| 22 | *PavNAC22* | FUN_010214-T1 | chr_2 | 25886369 | 25890307 | 1455 | 484 |
| 23 | *PavNAC23* | FUN_010291-T1 | chr_2 | 26380729 | 26386993 | 1956 | 651 |
| 24 | *PavNAC24* | FUN_010295-T1 | chr_2 | 26407008 | 26410945 | 1776 | 591 |
| 25 | *PavNAC25* | FUN_011686-T1 | chr_2 | 37035755 | 37037260 | 1191 | 396 |
| 26 | *PavNAC26* | FUN_011689-T1 | chr_2 | 37072392 | 37073417 | 663 | 220 |
| 27 | *PavNAC27* | FUN_011750-T1 | chr_2 | 37438818 | 37443959 | 1929 | 642 |
| 28 | *PavNAC28* | FUN_011751-T1 | chr_2 | 37464841 | 37467748 | 840 | 279 |
| 29 | *PavNAC29* | FUN_011753-T1 | chr_2 | 37470931 | 37474258 | 1383 | 460 |
| 30 | *PavNAC30* | FUN_011754-T1 | chr_2 | 37477970 | 37481727 | 1518 | 505 |
| 31 | *PavNAC31* | FUN_011755-T1 | chr_2 | 37483344 | 37486412 | 1350 | 494 |
| 32 | *PavNAC32* | FUN_011756-T1 | chr_2 | 37490972 | 37493272 | 1614 | 537 |
| 33 | *PavNAC33* | FUN_011761-T1 | chr_2 | 37495445 | 37498490 | 1416 | 471 |
| 34 | *PavNAC34* | FUN_011762-T1 | chr_2 | 37525537 | 37527820 | 1332 | 443 |
| 35 | *PavNAC35* | FUN_011763-T1 | chr_2 | 37528061 | 37530843 | 1047 | 348 |
| 36 | *PavNAC36* | FUN_011769-T1 | chr_2 | 37532988 | 37538851 | 1443 | 480 |
| 37 | *PavNAC37* | FUN_011771-T1 | chr_2 | 37536425 | 37538896 | 1311 | 436 |
| 38 | *PavNAC38* | FUN_011776-T1 | chr_2 | 37536425 | 37538896 | 1761 | 586 |
| 39 | *PavNAC39* | FUN_011777-T1 | chr_2 | 37556326 | 37559266 | 999 | 332 |
| 40 | *PavNAC40* | FUN_011788-T1 | chr_2 | 37559899 | 37562810 | 594 | 197 |
| 41 | *PavNAC41* | FUN_011789-T1 | chr_2 | 37588410 | 37596111 | 483 | 160 |
| 42 | *PavNAC42* | FUN_011791-T1 | chr_2 | 37598739 | 37601330 | 657 | 218 |
| 43 | *PavNAC43* | FUN_011792-T1 | chr_2 | 37664302 | 37665510 | 519 | 172 |
| 44 | *PavNAC44* | FUN_012665-T1 | chr_2 | 37667717 | 37668536 | 1005 | 334 |
| 45 | *PavNAC45* | FUN_013524-T1 | chr_2 | 37673154 | 37674598 | 1896 | 631 |
| 46 | *PavNAC46* | FUN_014972-T1 | chr_2 | 37676561 | 37677920 | 576 | 191 |
| 47 | *PavNAC47* | FUN_014991-T1 | chr_2 | 42458971 | 42460225 | 1356 | 451 |
| 48 | *PavNAC48* | FUN_015278-T1 | chr_3 | 5211749 | 5215831 | 1368 | 455 |
| 49 | *PavNAC49* | FUN_015419-T1 | chr_3 | 16295576 | 16296151 | 639 | 212 |
| 50 | *PavNAC50* | FUN_016205-T1 | chr_3 | 16422242 | 16424013 | 918 | 306 |
| 51 | *PavNAC51* | FUN_017426-T1 | chr_3 | 18479124 | 18483534 | 1059 | 352 |
| 52 | *PavNAC52* | FUN_017464-T1 | chr_3 | 19715757 | 19716870 | 1191 | 396 |
| 53 | *PavNAC53* | FUN_017683-T1 | chr_3 | 25877265 | 25879769 | 1170 | 389 |
| 54 | *PavNAC54* | FUN_017871-T1 | chr_3 | 33807826 | 33809515 | 714 | 237 |
| 55 | *PavNAC55* | FUN_018914-T1 | chr_3 | 34029087 | 34030598 | 963 | 320 |
| 56 | *PavNAC56* | FUN_019314-T1 | chr_3 | 35234134 | 35237765 | 483 | 160 |
| 57 | *PavNAC57* | FUN_019402-T1 | chr_3 | 36110334 | 36111696 | 654 | 217 |
| 58 | *PavNAC58* | FUN_032796-T1 | chr_4 | 2505002 | 2507027 | 909 | 302 |
| 59 | *PavNAC59* | FUN_032867-T1 | chr_4 | 3399020 | 3401916 | 1560 | 519 |
| 60 | *PavNAC60* | FUN_033007-T1 | chr_4 | 3454463 | 3457630 | 1116 | 371 |
| 61 | *PavNAC61* | FUN_033091-T1 | chr_4 | 7694576 | 7695977 | 1116 | 371 |
| 62 | *PavNAC62* | FUN_033150-T1 | chr_4 | 8187796 | 8189674 | 1065 | 354 |
| 63 | *PavNAC63* | FUN_033300-T1 | chr_4 | 9325376 | 9326799 | 969 | 322 |
| 64 | *PavNAC64* | FUN_033398-T1 | chr_4 | 9832605 | 9834024 | 975 | 324 |
| 65 | *PavNAC65* | FUN_033470-T1 | chr_4 | 10173847 | 10176960 | 1014 | 337 |
| 66 | *PavNAC66* | FUN_033490-T1 | chr_4 | 11158179 | 11160528 | 849 | 282 |
| 67 | *PavNAC67* | FUN_033497-T1 | chr_4 | 11842464 | 11846387 | 924 | 307 |
| 68 | *PavNAC68* | FUN_033498-T1 | chr_4 | 12329086 | 12332147 | 885 | 294 |
| 69 | *PavNAC69* | FUN_033856-T1 | chr_4 | 12464045 | 12465675 | 1077 | 358 |
| 70 | *PavNAC70* | FUN_033982-T1 | chr_4 | 12522509 | 12525212 | 1047 | 348 |
| 71 | *PavNAC71* | FUN_033984-T1 | chr_4 | 12528459 | 12529343 | 1086 | 361 |
| 72 | *PavNAC72* | FUN_034469-T1 | chr_4 | 15130481 | 15132193 | 1302 | 433 |
| 73 | *PavNAC73* | FUN_034470-T1 | chr_4 | 15990992 | 15992877 | 1944 | 647 |
| 74 | *PavNAC74* | FUN_034473-T1 | chr_4 | 16010260 | 16012585 | 1122 | 373 |
| 75 | *PavNAC75* | FUN_034475-T1 | chr_4 | 19946507 | 19948875 | 1137 | 378 |
| 76 | *PavNAC76* | FUN_034478-T1 | chr_4 | 19954535 | 19958517 | 1380 | 459 |
| 77 | *PavNAC77* | FUN_034543-T1 | chr_4 | 19978926 | 19980047 | 1137 | 378 |
| 78 | *PavNAC78* | FUN_034545-T1 | chr_4 | 20000821 | 20001957 | 1380 | 459 |
| 79 | *PavNAC79* | FUN_034798-T1 | chr_4 | 20027237 | 20029140 | 1680 | 559 |
| 80 | *PavNAC80* | FUN_034801-T1 | chr_4 | 20653448 | 20654584 | 1152 | 383 |
| 81 | *PavNAC81* | FUN_035257-T1 | chr_4 | 20669716 | 20671618 | 879 | 292 |
| 82 | *PavNAC82* | FUN_036080-T1 | chr_4 | 22885421 | 22890667 | 1461 | 486 |
| 83 | *PavNAC83* | FUN_036091-T1 | chr_4 | 22909962 | 22911684 | 1497 | 498 |
| 84 | *PavNAC84* | FUN_036243-T1 | chr_4 | 27299749 | 27301830 | 1380 | 459 |
| 85 | *PavNAC85* | FUN_025470-T1 | chr_5 | 18628153 | 18630514 | 1188 | 395 |
| 86 | *PavNAC86* | FUN_025496-T1 | chr_5 | 21010278 | 21013176 | 2268 | 755 |
| 87 | *PavNAC87* | FUN_025597-T1 | chr_5 | 24269734 | 24271321 | 708 | 235 |
| 88 | *PavNAC88* | FUN_026204-T1 | chr_5 | 28392631 | 28394007 | 1083 | 360 |
| 89 | *PavNAC89* | FUN_026453-T1 | chr_5 | 28557547 | 28561310 | 1065 | 354 |
| 90 | *PavNAC90* | FUN_026676-T1 | chr_5 | 29131529 | 29132407 | 786 | 261 |
| 91 | *PavNAC91* | FUN_027141-T1 | chr_5 | 32328487 | 32330332 | 1056 | 351 |
| 92 | *PavNAC92* | FUN_027142-T1 | chr_5 | 33763446 | 33765161 | 732 | 243 |
| 93 | *PavNAC93* | FUN_027146-T1 | chr_5 | 34892702 | 34894931 | 858 | 185 |
| 94 | *PavNAC94* | FUN_019403-T1 | chr_6 | 6331169 | 6332879 | 597 | 198 |
| 95 | *PavNAC95* | FUN_019405-T1 | chr_6 | 8989724 | 8990206 | 1056 | 351 |
| 96 | *PavNAC96* | FUN_019547-T1 | chr_6 | 9677003 | 9678034 | 993 | 330 |
| 97 | *PavNAC97* | FUN_019548-T1 | chr_6 | 9681930 | 9683835 | 1980 | 659 |
| 98 | *PavNAC98* | FUN_019561-T1 | chr_6 | 9690790 | 9692541 | 1050 | 349 |
| 99 | *PavNAC99* | FUN_019562-T1 | chr_6 | 10883762 | 10885493 | 993 | 330 |
| 100 | *PavNAC100* | FUN_019862-T1 | chr_6 | 10897647 | 10902940 | 990 | 329 |
| 101 | *PavNAC101* | FUN_019867-T1 | chr_6 | 10980602 | 10981744 | 816 | 271 |
| 102 | *PavNAC102* | FUN_019882-T1 | chr_6 | 10984706 | 10986435 | 1170 | 389 |
| 103 | *PavNAC103* | FUN_020951-T1 | chr_6 | 13679439 | 13680762 | 849 | 282 |
| 104 | *PavNAC104* | FUN_021693-T1 | chr_6 | 13726685 | 13728282 | 1074 | 357 |
| 105 | *PavNAC105* | FUN_024171-T1 | chr_6 | 13885989 | 13890601 | 543 | 180 |
| 106 | *PavNAC106* | FUN_024427-T1 | chr_6 | 23604481 | 23606043 | 1035 | 244 |
| 107 | *PavNAC107* | FUN_024846-T1 | chr_6 | 29397010 | 29399044 | 1185 | 394 |
| 108 | *PavNAC108* | FUN_036249-T1 | chr_7 | 3193191 | 3196750 | 1158 | 385 |
| 109 | *PavNAC109* | FUN_036581-T1 | chr_7 | 3298631 | 3303673 | 747 | 248 |
| 110 | *PavNAC110* | FUN_037748-T1 | chr_7 | 4986817 | 4988945 | 1665 | 554 |
| 111 | *PavNAC111* | FUN_037783-T1 | chr_7 | 5031058 | 5033296 | 1290 | 429 |
| 112 | *PavNAC112* | FUN_038023-T1 | chr_7 | 8064597 | 8066619 | 858 | 285 |
| 113 | *PavNAC113* | FUN_038721-T1 | chr_7 | 17996536 | 17999165 | 1020 | 339 |
| 114 | *PavNAC114* | FUN_039321-T1 | chr_7 | 18234015 | 18236938 | 1464 | 487 |
| 115 | *PavNAC115* | FUN_040030-T1 | chr_7 | 20090365 | 20091849 | 1284 | 427 |
| 116 | *PavNAC116* | FUN_040031-T3 | chr_7 | 27585954 | 27589627 | 1374 | 457 |
| 117 | *PavNAC117* | FUN_027248-T1 | chr_8 | 6561068 | 6564504 | 1299 | 432 |
| 118 | *PavNAC118* | FUN_027251-T1 | chr_8 | 6566748 | 6567763 | 1056 | 351 |
| 119 | *PavNAC119* | FUN_027252-T1 | chr_8 | 6577365 | 6578555 | 900 | 299 |
| 120 | *PavNAC120* | FUN_027825-T1 | chr_8 | 7413678 | 7416266 | 1149 | 382 |
| 121 | *PavNAC121* | FUN_028005-T1 | chr_8 | 7453870 | 7457370 | 1734 | 580 |
| 122 | *PavNAC122* | FUN_028385-T1 | chr_8 | 7459000 | 7461148 | 459 | 152 |
| 123 | *PavNAC123* | FUN_029005-T1 | chr_8 | 12071363 | 12073358 | 783 | 260 |
| 124 | *PavNAC124* | FUN_029038-T1 | chr_8 | 13602052 | 13606203 | 816 | 271 |
| 125 | *PavNAC125* | FUN_029305-T1 | chr_8 | 16669290 | 16670310 | 933 | 310 |
| 126 | *PavNAC126* | FUN_029359-T1 | chr_8 | 21517583 | 21519226 | 933 | 310 |
| 127 | *PavNAC127* | FUN_029385-T1 | chr_8 | 21798842 | 21800518 | 861 | 286 |
| 128 | *PavNAC128* | FUN_030360-T1 | chr_8 | 24098736 | 24099901 | 876 | 291 |
| 129 | *PavNAC129* | FUN_030930-T1 | chr_8 | 24584785 | 24585951 | 858 | 285 |
| 130 | *PavNAC130* | FUN_031953-T1 | chr_8 | 24746819 | 24747679 | 1098 | 365 |
| 131 | *PavNAC131* | FUN_032107-T1 | chr_8 | 31161341 | 31163454 | 954 | 317 |
| 132 | *PavNAC132* | FUN_032118-T1 | chr_8 | 34603848 | 34607439 | 1455 | 484 |
